# Rhodamine 6G hexa­chlorido­stannate(IV) acetonitrile disolvate

**DOI:** 10.1107/S1600536807066287

**Published:** 2007-12-18

**Authors:** Ramaiyer Venkatraman, Lungile Sitole, Frank R. Fronczek

**Affiliations:** a Department of Chemistry, Jackson State University, Jackson, MS 39217, USA; b Department of Chemistry, Louisiana State University, Baton Rouge, LA 70803-1804, USA

## Abstract

In the title compound, bis({6-ethylamino-10-[2-(methoxycarbonyl)phenyl]-2,7-dimethylxanthen-3-ylidene}ethanaminium) hexachloridotin(IV) acetonitrile disolvate, (C_27_H_29_N_2_O_3_)_2_[SnCl_6_]·2C_2_H_3_N, the octa­hedral SnCl_6_^2−^ anion lies on an inversion center. The xanthene ring system is essentially planar, with an average deviation of 0.020 Å, and the substituent benzene ring forms a dihedral angle of 85.89 (2)° with it. The Sn—Cl distances are in the range 2.4237 (3)–2.4454 (3) Å. There are N—H⋯Cl hydrogen bonds between SnCl_6_^2−^ ions and rhodamine 6G cations as well as π–π stacking inter­actions between rhodamine 6G cations (inter­planar distance of 3.827 Å).

## Related literature

For related literature, see: Bhagavthy *et al.* (1993 ▶); Fun *et al.* (1997 ▶); Herz (1974 ▶); Johnson & McGrane (1993 ▶); Liu *et al.* (1998 ▶); Nguyen & Meyer (1992 ▶); Wang *et al.* (1997 ▶). For the structure of the analogous ethyl ester as the chloride salt hydrate, see: Adhikesavalu *et al.* (2001 ▶).
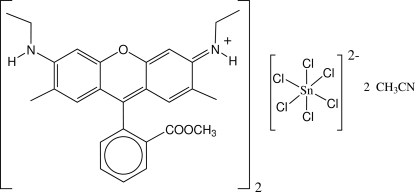


## Experimental

### 

#### Crystal data

(C_27_H_29_N_2_O_3_)_2_[SnCl_6_]·2C_2_H_3_N
*M* *_r_* = 1272.54
Triclinic, 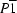

*a* = 9.7871 (10) Å
*b* = 11.7827 (11) Å
*c* = 13.2893 (12) Å
α = 80.583 (4)°
β = 77.309 (4)°
γ = 82.467 (4)°
*V* = 1467.7 (2) Å^3^
*Z* = 1
Mo *K*α radiation
μ = 0.76 mm^−1^
*T* = 90.0 (5) K
0.28 × 0.27 × 0.25 mm

#### Data collection

Nonius KappaCCD diffractometer with Oxford Cryostream
Absorption correction: multi-scan (*DENZO* and *SCALEPACK*; Otwinowski & Minor, 1997 ▶) *T*_min_ = 0.815, *T*_max_ = 0.832
54250 measured reflections
14412 independent reflections
12969 reflections with *I* > 2σ(*I*)
*R*_int_ = 0.024

#### Refinement

*R*[*F* ^2^ > 2σ(*F* ^2^)] = 0.029
*wR*(*F* ^2^) = 0.073
*S* = 1.03
14412 reflections
362 parameters
H atoms treated by a mixture of independent and constrained refinement
Δρ_max_ = 0.74 e Å^−3^
Δρ_min_ = −1.24 e Å^−3^

### 

Data collection: *COLLECT* (Nonius, 2000 ▶); cell refinement: *DENZO* and *SCALEPACK* (Otwinowski & Minor, 1997 ▶); data reduction: *DENZO* and *SCALEPACK*; program(s) used to solve structure: *SIR97* (Altomare *et al.*, 1999 ▶); program(s) used to refine structure: *SHELXL97* (Sheldrick, 1997 ▶); molecular graphics: *ORTEP-3 for Windows* (Farrugia, 1997 ▶); software used to prepare material for publication: *SHELXL97*.

## Supplementary Material

Crystal structure: contains datablocks global, I. DOI: 10.1107/S1600536807066287/pv2056sup1.cif

Structure factors: contains datablocks I. DOI: 10.1107/S1600536807066287/pv2056Isup2.hkl

Additional supplementary materials:  crystallographic information; 3D view; checkCIF report

## Figures and Tables

**Table 1:** Hydrogen-bond geometry (Å, °)

*D*—H⋯*A*	*D*—H	H⋯*A*	*D*⋯*A*	*D*—H⋯*A*
N1—H1N⋯Cl2	0.81 (2)	2.61 (2)	3.3644 (10)	156 (2)
N2—H2N⋯Cl1^i^	0.86 (2)	2.75 (2)	3.5603 (10)	159 (2)

Symmetry code: (i) 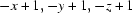.

**Fig. 1.**
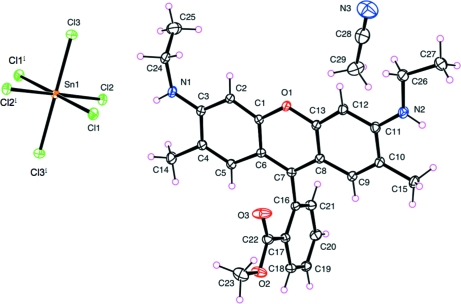
Numbering scheme and ellipsoids at the 50% level. (i = 1 - x, -y, -z).

**Fig. 2.**
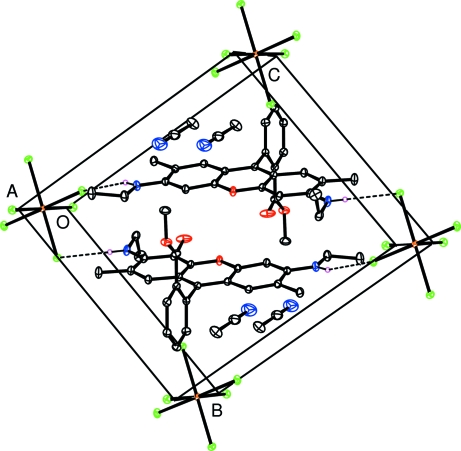
The unit cell, showing stacking of rhodamine cations and hydrogen bonding.

## supplementary crystallographic information

### Comment

The excellent photo-physical properties of rhodamines are well known (Herz, 1974; Johnson & McGrane, 1993; Nguyen & Meyer, 1992), and recently metal complexes of rhodamine 6 G have been reported by several authors (Bhagavthy *et al.*, 1993; Fun *et al.*, 1997; Wang *et al.*, 1997; Liu *et al.*, 1998). The aggregative properties of cationic species of the dye were observed to be dependent on the anionic environment created by the metal ions. We have synthesized a rhodamine 6 G derivative, 9-[2-methoxycarbonyl)phenyl]-3,6-bis(ethylamino)-2,7-dimethylxanthylium hexachlorotin(IV) diacetonitrile solvate, (I), the structure of which is presented in this paper.

The structure of (I) consists of discrete SnCl_6_^2-^ anions lying on inversion centers, rhodamine 6 G cations and acetonitrile solvent molecules (Fig. 1). The xanthene ring of the cation is planar within an average deviation of 0.020 Å (maximum deviation is 0.045 (1) Å for C4), and the phenyl ring forms a dihedral angle of 85.89 (2)° with it. The C—N distances N1—C24 and N2—C26 are normal for this type of single bond, whereas C3—N1 and C11—N2 are much shorter, showing partial double-bond character; details are in the Table. A similar trend is observed in the other rhodamine 6 G cations (Wang *et al.*, 1997; Liu *et al.*, 1998).

Both hydrogen bonding between cations and anions and π-π stacking interactions between rhodamine 6 G cations exist. Parallel rhodamine ions related by the inversion center have an interplanar distance of 3.827 Å (Fig. 2), and are slipped such that their O1—C13 bonds exactly overlap.

### Experimental

Diphenyl tin dichloride (0.344 g, 1 mmol) was dissolved in 20 ml me thanol, and then 20 ml of methanol solution of rhodamine 6 G (0.479 g, 1 mmol) was added. The bright red solution was refluxed for 1 hr, whereafter red brown solid were obtained on cooling. Suitable size crystals were obtained by the recrystallization at room temperature from acetonitrile (yield *ca* 85%).

### Refinement

H atoms were placed in idealized positions with C—H distances at 0.99, 0.98 and 0.95 Å for CH_2_, CH_3_ and aromatic CH groups, respectively using a riding model. *U*_iso_ for H was assigned as 1.2 times *U*_eq_ of the attached C atoms (1.5 for methyl); a torsional parameter was refined for each methyl group. The largest positive and negative residual density peaks were located within 1 Å of the Sn1 position.

### Crystal data

**Table:**

(C_27_H_29_N_2_O_3_)_2_[SnCl_6_]·2C_2_H_3_N	*Z* = 1
*M**_r_* = 1272.54	*F*_000_ = 654
Triclinic, *P*1	*D*_x_ = 1.440 Mg m^−^^3^
Hall symbol: -P 1	Mo *K*α radiation λ = 0.71073 Å
*a* = 9.7871 (10) Å	Cell parameters from 13141 reflections
*b* = 11.7827 (11) Å	θ = 2.5–36.8º
*c* = 13.2893 (12) Å	µ = 0.76 mm^−^^1^
α = 80.583 (4)º	*T* = 90.0 (5) K
β = 77.309 (4)º	Fragment, red-orange
γ = 82.467 (4)º	0.28 × 0.27 × 0.25 mm
*V* = 1467.7 (2) Å^3^	

### Data collection

**Table:**

Nonius KappaCCD diffractometer with Oxford Cryostream	14412 independent reflections
Radiation source: fine-focus sealed tube	12969 reflections with *I* > 2σ(*I*)
Monochromator: graphite	*R*_int_ = 0.024
*T* = 90.0(5) K	θ_max_ = 36.8º
ω scans with κ offsets	θ_min_ = 2.7º
Absorption correction: multi-scan(DENZO and SCALEPACK; Otwinowski & Minor, 1997)	*h* = −16→16
*T*_min_ = 0.815, *T*_max_ = 0.832	*k* = −19→19
54250 measured reflections	*l* = −22→21

### Refinement

**Table:**

Refinement on *F*^2^	Hydrogen site location: inferred from neighbouring sites
Least-squares matrix: full	H atoms treated by a mixture of independent and constrained refinement
*R*[*F*^2^ > 2σ(*F*^2^)] = 0.029	*w* = 1/[σ^2^(*F*_o_^2^) + (0.0303*P*)^2^ + 0.821*P*] where *P* = (*F*_o_^2^ + 2*F*_c_^2^)/3
*wR*(*F*^2^) = 0.073	(Δ/σ)_max_ < 0.001
*S* = 1.03	Δρ_max_ = 0.74 e Å^−^^3^
14412 reflections	Δρ_min_ = −1.24 e Å^−^^3^
362 parameters	Extinction correction: SHELXL, Fc^*^=kFc[1+0.001xFc^2^λ^3^/sin(2θ)]^-1/4^
Primary atom site location: structure-invariant direct methods	Extinction coefficient: 0.0053 (5)
Secondary atom site location: difference Fourier map	

### Special details

**Table:**

Geometry. All e.s.d.'s (except the e.s.d. in the dihedral angle between two l.s. planes) are estimated using the full covariance matrix. The cell e.s.d.'s are taken into account individually in the estimation of e.s.d.'s in distances, angles and torsion angles; correlations between e.s.d.'s in cell parameters are only used when they are defined by crystal symmetry. An approximate (isotropic) treatment of cell e.s.d.'s is used for estimating e.s.d.'s involving l.s. planes.
Refinement. Refinement of *F*^2^ against ALL reflections. The weighted *R*-factor *wR* and goodness of fit *S* are based on *F*^2^, conventional *R*-factors *R* are based on *F*, with *F* set to zero for negative *F*^2^. The threshold expression of *F*^2^ > σ(*F*^2^) is used only for calculating *R*-factors(gt) *etc*. and is not relevant to the choice of reflections for refinement. *R*-factors based on *F*^2^ are statistically about twice as large as those based on *F*, and *R*- factors based on ALL data will be even larger.

### Fractional atomic coordinates and isotropic or equivalent isotropic displacement parameters (Å^2^)

**Table:**

	*x*	*y*	*z*	*U*_iso_*/*U*_eq_	
Sn1	0.5000	0.0000	0.0000	0.00926 (3)	
Cl1	0.53900 (3)	0.04133 (2)	0.164917 (19)	0.01502 (5)	
Cl2	0.51522 (3)	0.20275 (2)	−0.07554 (2)	0.01362 (4)	
Cl3	0.24802 (3)	0.04038 (2)	0.05732 (2)	0.01381 (4)	
O1	0.40633 (8)	0.58975 (7)	0.38427 (6)	0.01354 (13)	
O2	1.06811 (9)	0.46854 (8)	0.36352 (7)	0.01853 (15)	
O3	0.83375 (10)	0.47563 (9)	0.39044 (9)	0.0272 (2)	
N1	0.42673 (10)	0.33594 (9)	0.13842 (8)	0.01642 (17)	
H1N	0.4731 (19)	0.3059 (16)	0.0897 (14)	0.020*	
N2	0.35159 (10)	0.83221 (9)	0.64389 (7)	0.01542 (16)	
H2N	0.3892 (19)	0.8743 (16)	0.6750 (14)	0.019*	
C1	0.48174 (10)	0.54161 (9)	0.30004 (8)	0.01118 (15)	
C2	0.41464 (10)	0.46614 (9)	0.26289 (8)	0.01275 (16)	
H2	0.3206	0.4516	0.2948	0.015*	
C3	0.48671 (11)	0.41085 (9)	0.17721 (8)	0.01291 (16)	
C4	0.63032 (11)	0.43418 (9)	0.12992 (8)	0.01360 (16)	
C5	0.69115 (11)	0.51151 (9)	0.16815 (8)	0.01310 (16)	
H5	0.7846	0.5275	0.1359	0.016*	
C6	0.62049 (10)	0.56913 (9)	0.25423 (8)	0.01122 (15)	
C7	0.68061 (10)	0.64746 (8)	0.29612 (8)	0.01058 (15)	
C8	0.60111 (10)	0.69685 (9)	0.38325 (8)	0.01108 (15)	
C9	0.65144 (10)	0.77664 (9)	0.43315 (8)	0.01170 (15)	
H9	0.7429	0.8005	0.4054	0.014*	
C10	0.57244 (11)	0.82019 (9)	0.51976 (8)	0.01222 (15)	
C11	0.43234 (11)	0.78594 (9)	0.56098 (8)	0.01258 (16)	
C12	0.38037 (11)	0.70714 (9)	0.51364 (8)	0.01331 (16)	
H12	0.2889	0.6832	0.5408	0.016*	
C13	0.46414 (10)	0.66475 (9)	0.42693 (8)	0.01159 (15)	
C14	0.70941 (13)	0.37222 (11)	0.04109 (10)	0.0197 (2)	
H14A	0.8074	0.3907	0.0232	0.030*	
H14B	0.6655	0.3969	−0.0195	0.030*	
H14C	0.7069	0.2886	0.0616	0.030*	
C15	0.62856 (12)	0.90417 (10)	0.57043 (9)	0.01553 (17)	
H15A	0.7239	0.9186	0.5326	0.023*	
H15B	0.6310	0.8716	0.6428	0.023*	
H15C	0.5674	0.9770	0.5689	0.023*	
C16	0.82316 (10)	0.68456 (8)	0.24669 (8)	0.01064 (15)	
C17	0.94862 (10)	0.62467 (9)	0.27097 (8)	0.01147 (15)	
C18	1.07793 (11)	0.66730 (9)	0.22341 (8)	0.01360 (16)	
H18	1.1625	0.6273	0.2402	0.016*	
C19	1.08333 (11)	0.76787 (9)	0.15174 (8)	0.01417 (17)	
H19	1.1713	0.7963	0.1196	0.017*	
C20	0.95942 (12)	0.82636 (10)	0.12744 (9)	0.01498 (17)	
H20	0.9629	0.8948	0.0784	0.018*	
C21	0.82987 (11)	0.78526 (9)	0.17463 (8)	0.01412 (17)	
H21	0.7457	0.8260	0.1576	0.017*	
C22	0.94161 (11)	0.51676 (9)	0.34679 (9)	0.01400 (17)	
C23	1.06484 (13)	0.36159 (11)	0.43498 (10)	0.0211 (2)	
H23A	0.9925	0.3717	0.4976	0.032*	
H23B	1.1569	0.3406	0.4543	0.032*	
H23C	1.0429	0.3000	0.4014	0.032*	
C24	0.28833 (12)	0.29543 (10)	0.18084 (9)	0.01633 (18)	
H24A	0.2628	0.3019	0.2562	0.020*	
H24B	0.2931	0.2127	0.1733	0.020*	
C25	0.17375 (14)	0.36257 (13)	0.12788 (12)	0.0258 (2)	
H25A	0.1665	0.4443	0.1367	0.039*	
H25B	0.0836	0.3314	0.1593	0.039*	
H25C	0.1973	0.3554	0.0535	0.039*	
C26	0.20472 (12)	0.81093 (11)	0.68565 (9)	0.0185 (2)	
H26A	0.2000	0.7296	0.7190	0.022*	
H26B	0.1527	0.8234	0.6281	0.022*	
C27	0.13662 (13)	0.89136 (12)	0.76511 (10)	0.0232 (2)	
H27A	0.1871	0.8779	0.8227	0.035*	
H27B	0.0382	0.8763	0.7920	0.035*	
H27C	0.1406	0.9718	0.7319	0.035*	
N3	−0.01218 (17)	0.87440 (15)	0.42927 (13)	0.0429 (4)	
C28	0.09893 (16)	0.88240 (12)	0.37996 (11)	0.0259 (2)	
C29	0.23901 (15)	0.89178 (14)	0.31671 (11)	0.0267 (3)	
H29A	0.2723	0.8203	0.2862	0.040*	
H29B	0.3031	0.9042	0.3603	0.040*	
H29C	0.2364	0.9571	0.2609	0.040*	

### Atomic displacement parameters (Å^2^)

**Table:**

	*U*^11^	*U*^22^	*U*^33^	*U*^12^	*U*^13^	*U*^23^
Sn1	0.01002 (4)	0.00916 (4)	0.00947 (4)	−0.00130 (3)	−0.00229 (3)	−0.00309 (3)
Cl1	0.01755 (10)	0.01736 (11)	0.01246 (10)	−0.00091 (8)	−0.00530 (8)	−0.00629 (8)
Cl2	0.01605 (10)	0.01023 (9)	0.01489 (10)	−0.00222 (7)	−0.00319 (8)	−0.00189 (7)
Cl3	0.01070 (9)	0.01542 (10)	0.01489 (10)	−0.00084 (7)	−0.00195 (7)	−0.00214 (8)
O1	0.0112 (3)	0.0151 (3)	0.0155 (3)	−0.0041 (2)	0.0009 (2)	−0.0078 (3)
O2	0.0122 (3)	0.0194 (4)	0.0205 (4)	0.0012 (3)	−0.0034 (3)	0.0050 (3)
O3	0.0140 (4)	0.0235 (4)	0.0401 (6)	−0.0072 (3)	−0.0080 (4)	0.0150 (4)
N1	0.0148 (4)	0.0163 (4)	0.0198 (4)	−0.0041 (3)	0.0003 (3)	−0.0103 (3)
N2	0.0155 (4)	0.0181 (4)	0.0135 (4)	−0.0043 (3)	0.0004 (3)	−0.0073 (3)
C1	0.0097 (3)	0.0108 (4)	0.0131 (4)	−0.0009 (3)	−0.0008 (3)	−0.0041 (3)
C2	0.0104 (4)	0.0126 (4)	0.0162 (4)	−0.0023 (3)	−0.0012 (3)	−0.0058 (3)
C3	0.0126 (4)	0.0116 (4)	0.0155 (4)	−0.0019 (3)	−0.0024 (3)	−0.0047 (3)
C4	0.0120 (4)	0.0142 (4)	0.0150 (4)	−0.0018 (3)	−0.0005 (3)	−0.0058 (3)
C5	0.0109 (4)	0.0143 (4)	0.0143 (4)	−0.0017 (3)	−0.0002 (3)	−0.0054 (3)
C6	0.0094 (3)	0.0113 (4)	0.0132 (4)	−0.0014 (3)	−0.0015 (3)	−0.0034 (3)
C7	0.0092 (3)	0.0106 (4)	0.0119 (4)	−0.0011 (3)	−0.0016 (3)	−0.0022 (3)
C8	0.0106 (4)	0.0112 (4)	0.0118 (4)	−0.0019 (3)	−0.0017 (3)	−0.0026 (3)
C9	0.0116 (4)	0.0114 (4)	0.0128 (4)	−0.0022 (3)	−0.0026 (3)	−0.0026 (3)
C10	0.0133 (4)	0.0121 (4)	0.0123 (4)	−0.0023 (3)	−0.0031 (3)	−0.0030 (3)
C11	0.0136 (4)	0.0126 (4)	0.0115 (4)	−0.0019 (3)	−0.0010 (3)	−0.0032 (3)
C12	0.0129 (4)	0.0138 (4)	0.0135 (4)	−0.0039 (3)	0.0007 (3)	−0.0052 (3)
C13	0.0112 (4)	0.0115 (4)	0.0127 (4)	−0.0025 (3)	−0.0015 (3)	−0.0038 (3)
C14	0.0171 (5)	0.0222 (5)	0.0207 (5)	−0.0045 (4)	0.0030 (4)	−0.0129 (4)
C15	0.0174 (4)	0.0168 (4)	0.0149 (4)	−0.0039 (3)	−0.0038 (3)	−0.0067 (3)
C16	0.0095 (3)	0.0114 (4)	0.0114 (4)	−0.0020 (3)	−0.0014 (3)	−0.0028 (3)
C17	0.0099 (4)	0.0109 (4)	0.0135 (4)	−0.0015 (3)	−0.0016 (3)	−0.0020 (3)
C18	0.0097 (4)	0.0142 (4)	0.0168 (4)	−0.0018 (3)	−0.0015 (3)	−0.0029 (3)
C19	0.0122 (4)	0.0149 (4)	0.0153 (4)	−0.0037 (3)	−0.0001 (3)	−0.0034 (3)
C20	0.0152 (4)	0.0147 (4)	0.0144 (4)	−0.0045 (3)	−0.0019 (3)	0.0005 (3)
C21	0.0121 (4)	0.0140 (4)	0.0158 (4)	−0.0018 (3)	−0.0031 (3)	−0.0002 (3)
C22	0.0124 (4)	0.0128 (4)	0.0167 (4)	−0.0012 (3)	−0.0042 (3)	−0.0002 (3)
C23	0.0200 (5)	0.0197 (5)	0.0199 (5)	0.0023 (4)	−0.0043 (4)	0.0049 (4)
C24	0.0158 (4)	0.0139 (4)	0.0206 (5)	−0.0046 (3)	−0.0018 (4)	−0.0065 (4)
C25	0.0188 (5)	0.0259 (6)	0.0337 (7)	−0.0029 (4)	−0.0078 (5)	−0.0030 (5)
C26	0.0152 (4)	0.0217 (5)	0.0186 (5)	−0.0044 (4)	0.0023 (4)	−0.0088 (4)
C27	0.0178 (5)	0.0290 (6)	0.0230 (5)	−0.0010 (4)	0.0017 (4)	−0.0134 (5)
N3	0.0356 (7)	0.0410 (8)	0.0433 (8)	−0.0075 (6)	0.0048 (6)	0.0053 (7)
C28	0.0311 (6)	0.0223 (6)	0.0233 (6)	−0.0063 (5)	−0.0045 (5)	0.0009 (4)
C29	0.0252 (6)	0.0370 (7)	0.0190 (5)	−0.0098 (5)	−0.0050 (4)	−0.0010 (5)

### Geometric parameters (Å, °)

**Table:**

Sn1—Cl3	2.4237 (3)	C14—H14A	0.9800
Sn1—Cl3^i^	2.4237 (3)	C14—H14B	0.9800
Sn1—Cl1^i^	2.4396 (3)	C14—H14C	0.9800
Sn1—Cl1	2.4396 (3)	C15—H15A	0.9800
Sn1—Cl2^i^	2.4454 (3)	C15—H15B	0.9800
Sn1—Cl2	2.4454 (3)	C15—H15C	0.9800
O1—C1	1.3618 (12)	C16—C21	1.3966 (15)
O1—C13	1.3632 (12)	C16—C17	1.4080 (14)
O2—C22	1.3417 (13)	C17—C18	1.4017 (14)
O2—C23	1.4479 (15)	C17—C22	1.4865 (15)
O3—C22	1.2078 (14)	C18—C19	1.3925 (15)
N1—C3	1.3443 (13)	C18—H18	0.9500
N1—C24	1.4583 (15)	C19—C20	1.3893 (16)
N1—H1N	0.81 (2)	C19—H19	0.9500
N2—C11	1.3553 (13)	C20—C21	1.3955 (15)
N2—C26	1.4613 (15)	C20—H20	0.9500
N2—H2N	0.86 (2)	C21—H21	0.9500
C1—C2	1.3802 (14)	C23—H23A	0.9800
C1—C6	1.4169 (14)	C23—H23B	0.9800
C2—C3	1.4099 (14)	C23—H23C	0.9800
C2—H2	0.9500	C24—C25	1.5201 (18)
C3—C4	1.4500 (15)	C24—H24A	0.9900
C4—C5	1.3673 (14)	C24—H24B	0.9900
C4—C14	1.5027 (15)	C25—H25A	0.9800
C5—C6	1.4252 (14)	C25—H25B	0.9800
C5—H5	0.9500	C25—H25C	0.9800
C6—C7	1.4008 (14)	C26—C27	1.5198 (16)
C7—C8	1.4109 (14)	C26—H26A	0.9900
C7—C16	1.4941 (14)	C26—H26B	0.9900
C8—C13	1.4128 (14)	C27—H27A	0.9800
C8—C9	1.4266 (14)	C27—H27B	0.9800
C9—C10	1.3727 (14)	C27—H27C	0.9800
C9—H9	0.9500	N3—C28	1.146 (2)
C10—C11	1.4442 (15)	C28—C29	1.450 (2)
C10—C15	1.5024 (14)	C29—H29A	0.9800
C11—C12	1.4043 (14)	C29—H29B	0.9800
C12—C13	1.3839 (14)	C29—H29C	0.9800
C12—H12	0.9500		
			
Cl3—Sn1—Cl3^i^	180.0	H14A—C14—H14C	109.5
Cl3—Sn1—Cl1^i^	89.363 (10)	H14B—C14—H14C	109.5
Cl3^i^—Sn1—Cl1^i^	90.638 (10)	C10—C15—H15A	109.5
Cl3—Sn1—Cl1	90.638 (10)	C10—C15—H15B	109.5
Cl3^i^—Sn1—Cl1	89.362 (10)	H15A—C15—H15B	109.5
Cl1^i^—Sn1—Cl1	180.0	C10—C15—H15C	109.5
Cl3—Sn1—Cl2^i^	90.291 (10)	H15A—C15—H15C	109.5
Cl3^i^—Sn1—Cl2^i^	89.709 (10)	H15B—C15—H15C	109.5
Cl1^i^—Sn1—Cl2^i^	90.829 (10)	C21—C16—C17	119.27 (9)
Cl1—Sn1—Cl2^i^	89.170 (10)	C21—C16—C7	117.43 (9)
Cl3—Sn1—Cl2	89.709 (10)	C17—C16—C7	123.29 (9)
Cl3^i^—Sn1—Cl2	90.290 (10)	C18—C17—C16	119.71 (9)
Cl1^i^—Sn1—Cl2	89.171 (11)	C18—C17—C22	121.02 (9)
Cl1—Sn1—Cl2	90.830 (10)	C16—C17—C22	119.27 (9)
Cl2^i^—Sn1—Cl2	180.0	C19—C18—C17	120.52 (10)
C1—O1—C13	120.30 (8)	C19—C18—H18	119.7
C22—O2—C23	114.57 (9)	C17—C18—H18	119.7
C3—N1—C24	126.24 (9)	C20—C19—C18	119.63 (10)
C3—N1—H1N	118.0 (13)	C20—C19—H19	120.2
C24—N1—H1N	115.6 (13)	C18—C19—H19	120.2
C11—N2—C26	123.53 (9)	C19—C20—C21	120.47 (10)
C11—N2—H2N	118.5 (12)	C19—C20—H20	119.8
C26—N2—H2N	118.0 (12)	C21—C20—H20	119.8
O1—C1—C2	116.05 (9)	C20—C21—C16	120.40 (10)
O1—C1—C6	121.03 (9)	C20—C21—H21	119.8
C2—C1—C6	122.93 (9)	C16—C21—H21	119.8
C1—C2—C3	119.50 (9)	O3—C22—O2	122.39 (10)
C1—C2—H2	120.3	O3—C22—C17	124.24 (10)
C3—C2—H2	120.3	O2—C22—C17	113.37 (9)
N1—C3—C2	121.95 (10)	O2—C23—H23A	109.5
N1—C3—C4	118.77 (9)	O2—C23—H23B	109.5
C2—C3—C4	119.29 (9)	H23A—C23—H23B	109.5
C5—C4—C3	119.00 (9)	O2—C23—H23C	109.5
C5—C4—C14	121.49 (10)	H23A—C23—H23C	109.5
C3—C4—C14	119.51 (9)	H23B—C23—H23C	109.5
C4—C5—C6	122.88 (9)	N1—C24—C25	113.19 (10)
C4—C5—H5	118.6	N1—C24—H24A	108.9
C6—C5—H5	118.6	C25—C24—H24A	108.9
C7—C6—C1	119.38 (9)	N1—C24—H24B	108.9
C7—C6—C5	124.23 (9)	C25—C24—H24B	108.9
C1—C6—C5	116.38 (9)	H24A—C24—H24B	107.8
C6—C7—C8	118.96 (9)	C24—C25—H25A	109.5
C6—C7—C16	121.31 (9)	C24—C25—H25B	109.5
C8—C7—C16	119.63 (9)	H25A—C25—H25B	109.5
C7—C8—C13	119.28 (9)	C24—C25—H25C	109.5
C7—C8—C9	124.03 (9)	H25A—C25—H25C	109.5
C13—C8—C9	116.67 (9)	H25B—C25—H25C	109.5
C10—C9—C8	122.33 (9)	N2—C26—C27	110.50 (10)
C10—C9—H9	118.8	N2—C26—H26A	109.5
C8—C9—H9	118.8	C27—C26—H26A	109.5
C9—C10—C11	119.04 (9)	N2—C26—H26B	109.5
C9—C10—C15	121.00 (9)	C27—C26—H26B	109.5
C11—C10—C15	119.95 (9)	H26A—C26—H26B	108.1
N2—C11—C12	120.82 (10)	C26—C27—H27A	109.5
N2—C11—C10	119.37 (9)	C26—C27—H27B	109.5
C12—C11—C10	119.80 (9)	H27A—C27—H27B	109.5
C13—C12—C11	119.16 (9)	C26—C27—H27C	109.5
C13—C12—H12	120.4	H27A—C27—H27C	109.5
C11—C12—H12	120.4	H27B—C27—H27C	109.5
O1—C13—C12	115.99 (9)	N3—C28—C29	179.32 (18)
O1—C13—C8	121.03 (9)	C28—C29—H29A	109.5
C12—C13—C8	122.98 (9)	C28—C29—H29B	109.5
C4—C14—H14A	109.5	H29A—C29—H29B	109.5
C4—C14—H14B	109.5	C28—C29—H29C	109.5
H14A—C14—H14B	109.5	H29A—C29—H29C	109.5
C4—C14—H14C	109.5	H29B—C29—H29C	109.5
			
C13—O1—C1—C2	179.54 (9)	C9—C10—C11—C12	1.40 (15)
C13—O1—C1—C6	−0.75 (15)	C15—C10—C11—C12	−179.79 (10)
O1—C1—C2—C3	−178.69 (9)	N2—C11—C12—C13	177.80 (10)
C6—C1—C2—C3	1.61 (16)	C10—C11—C12—C13	−0.92 (16)
C24—N1—C3—C2	−4.43 (18)	C1—O1—C13—C12	−178.66 (9)
C24—N1—C3—C4	174.88 (11)	C1—O1—C13—C8	1.91 (15)
C1—C2—C3—N1	179.50 (10)	C11—C12—C13—O1	−179.22 (9)
C1—C2—C3—C4	0.18 (16)	C11—C12—C13—C8	0.19 (16)
N1—C3—C4—C5	179.10 (11)	C7—C8—C13—O1	−2.03 (15)
C2—C3—C4—C5	−1.56 (16)	C9—C8—C13—O1	179.45 (9)
N1—C3—C4—C14	−1.55 (16)	C7—C8—C13—C12	178.58 (10)
C2—C3—C4—C14	177.78 (11)	C9—C8—C13—C12	0.06 (15)
C3—C4—C5—C6	1.23 (17)	C6—C7—C16—C21	93.07 (12)
C14—C4—C5—C6	−178.10 (11)	C8—C7—C16—C21	−83.31 (12)
O1—C1—C6—C7	−0.26 (15)	C6—C7—C16—C17	−88.32 (13)
C2—C1—C6—C7	179.42 (10)	C8—C7—C16—C17	95.30 (12)
O1—C1—C6—C5	178.39 (9)	C21—C16—C17—C18	0.58 (15)
C2—C1—C6—C5	−1.93 (15)	C7—C16—C17—C18	−178.01 (9)
C4—C5—C6—C7	179.04 (10)	C21—C16—C17—C22	−179.32 (9)
C4—C5—C6—C1	0.46 (16)	C7—C16—C17—C22	2.09 (15)
C1—C6—C7—C8	0.11 (15)	C16—C17—C18—C19	−0.52 (15)
C5—C6—C7—C8	−178.42 (10)	C22—C17—C18—C19	179.38 (10)
C1—C6—C7—C16	−176.29 (9)	C17—C18—C19—C20	0.11 (16)
C5—C6—C7—C16	5.18 (16)	C18—C19—C20—C21	0.25 (16)
C6—C7—C8—C13	1.00 (15)	C19—C20—C21—C16	−0.19 (16)
C16—C7—C8—C13	177.46 (9)	C17—C16—C21—C20	−0.23 (15)
C6—C7—C8—C9	179.40 (10)	C7—C16—C21—C20	178.44 (10)
C16—C7—C8—C9	−4.14 (15)	C23—O2—C22—O3	2.09 (17)
C7—C8—C9—C10	−177.98 (10)	C23—O2—C22—C17	−178.43 (10)
C13—C8—C9—C10	0.46 (15)	C18—C17—C22—O3	179.92 (12)
C8—C9—C10—C11	−1.18 (15)	C16—C17—C22—O3	−0.18 (17)
C8—C9—C10—C15	−179.97 (10)	C18—C17—C22—O2	0.46 (15)
C26—N2—C11—C12	−4.18 (17)	C16—C17—C22—O2	−179.64 (9)
C26—N2—C11—C10	174.54 (10)	C3—N1—C24—C25	96.39 (14)
C9—C10—C11—N2	−177.33 (10)	C11—N2—C26—C27	−169.80 (11)
C15—C10—C11—N2	1.48 (15)		

Symmetry codes: (i) −*x*+1, −*y*, −*z*.

### Hydrogen-bond geometry (Å, °)

**Table:**

*D*—H···*A*	*D*—H	H···*A*	*D*···*A*	*D*—H···*A*
N1—H1N···Cl2	0.81 (2)	2.61 (2)	3.3644 (10)	156 (2)
N2—H2N···Cl1^ii^	0.86 (2)	2.75 (2)	3.5603 (10)	159 (2)

Symmetry codes: (ii) −*x*+1, −*y*+1, −*z*+1.
